# Effect of Qinbai Qingfei Concentrated Pellets on substance P and neutral endopeptidase of rats with post-infectious cough

**DOI:** 10.1186/s12906-020-03081-5

**Published:** 2020-09-22

**Authors:** Weigang Jia, Wei Wang, Rui Li, Quanyu Zhou, Ying Qu, Yumei Jia, Zhiheng Zhang, Chengwei Wan, Wanwan Zhang

**Affiliations:** 1 Department of Emergency, Nangang Branch of Heilongjiang Academy of Traditional Chinese Medicine, Harbin, 150001 China; 2 Department of Orthopedics, Heilongjiang Provincial Hospital, Harbin, 150036 China; 3 The First Arrached Hostopal of Hebei Norruers Institlte, Zhangjiakou, 075000 China; 4 Gushi County Hospital of Traditional Chinese Medicine, Gushi, 465200 China

**Keywords:** Qinbai Qingfei concentrated pellet, Post-infectious cough, Inflammation, Oxidative stress, Substance P

## Abstract

**Background:**

In recent years, it has been reported that Qinbai Qingfei Concentrated Pellet (QQCP) has the effect of relieving cough and reducing sputum. However, the therapeutic potentials of QQCP on post-infectious cough (PIC) rat models has not been elucidated. So the current study was aimed to scientifically validate the efficacy of QQCP in post infectious cough.

**Methods:**

All rats were exposed to sawdust and cigarette smokes for 10 days, and intratracheal lipopolysaccharide (LPS) and capsaicin aerosols. Rats were treated with QQCP at dose of 80, 160, 320 mg/kg. Cough frequency was monitored twice a day for 10 days after drug administration. Inflammatory cell infiltration was determined by ELISA. Meanwhile, the histopathology of lung tissue and bronchus in rats were evaluated by hematoxylin-eosin staining (H&E). Neurogenetic inflammation were measured by ELISA and qRT-PCR.

**Results:**

QQCP dose-dependently decreased the cough frequency and the release of pro-inflammatory cytokines TNF-α, IL-1β, IL-6 and IL-8, but exerted the opposite effects on the secretion of anti-inflammatory cytokines IL-10 and IL-13 in BALF and serum of PIC rats. The oxidative burden was effectively ameliorated in QQCP-treated PIC rats as there were declines in Malondialdehyde (MDA) content and increases in Superoxide dismutase (SOD) activity in the serum and lung tissue. In addition, QQCP blocked inflammatory cell infiltration into the lung as evidenced by the reduced number of total leukocytes and the portion of neutrophils in the broncho - alveolar lavage fluid (BALF) as well as the alleviated lung damage. Furthermore, QQCP considerable reversed the neurogenetic inflammation caused by PIC through elevating neutral endopeptidase (NEP) activity and reducing Substance P (SP) and Calcitonin gene related peptide (CGRP) expression in BALF, serum and lung tissue.

**Conclusions:**

Our study indicated that QQCP demonstrated a protective role of PIC and may be a potential therapeutic target of PIC.

## Background

Cough is a common symptom of respiratory tract infection, which is caused by chemical or mechanical induction of sensory nerve receptors presented in the pharynx, larynx and bronchi [1]. Based on the duration, cough has been divided into acute cough (< 3 weeks), subacute cough (3–8 weeks) and chronic cough (> 8 weeks) [2]. Post-infectious cough (PIC), also known as post-cold cough, is identified as a type of subacute cough and accounts for 11–25% of chronic cough [3]. The pathogenesis of PIC is complicated, it could occur due to widespread airway inflammation, cough hypersensitivity and extensive disruption of epithelial integrity [4, 5]. Evidence has indicated that the frequency of PIC increased from 25 to 50% during outbursts of atypical pathogenic infections [6]. To date, there are few valid approaches available to combat PIC. Current therapy options such as antibiotics, antitussive drugs and corticosteroids improve cough, however, the outcomes of the current medicines are not satisfactory due to undesirable side effects. Hence, it is worthy to develop an effective alternative therapeutic agents for PIC treatment.

Traditional Chinese medicine (TCM) has been demonstrated to exert a pivotal role in the treatment of infectious diseases. For instance, *Pistacia weinmannifolia J. Poisson ex Franch* has been reported to effectively suppress the pulmonary inflammation stimulated by cigarette smoke (CS) and lipopolysaccharide (LPS) [7]. *Schisandra sphenanthera Rehd. et Wils*, a traditional polyherbal mixture used in cough, could effectively decline cough frequency and pulmonary inflammation in CS-evoked cough [8]. Further support for this was evidenced by recent researches which declared a significant inhibition in pulmonary inflammation following CS exposure after *Mahuang Tang* treatment [9]. Zhao et al. found that the efficacy of indole alkaloids against PIC was shown in the down-regulation of inflammatory cells and the balance of antioxidants [10]. The effects of Qufeng Xuanfei decoction in animal model of PIC were evaluated using lung pathology, cell counts and cell differentials in bronchoalveolar lavage (BAL), neurokinins A and B and calcitonin gene-related peptide (CGRP) [11]. Qinbai, extracted from natural sources, which is the first TCM used for *Mycoplasma* pneumonia (*M. pneumonia*) infections in China [12]. Several investigations have indicated that Qinbai not only possesses anti-*M. pneumonia* activity, but also owns intense protective effect on lung epithelial cells [12, 13]. Qinbai Qingfei Concentrated Pellet (QQCP) was developed by Heilongjiang Academy of Chinese Medical Sciences and was the first new drug approved by the State Food and Drug Administration (SFDA) (2004 L04185). It comprises 6 ingredients: *Scutellaria baicalensis* Georgi (major chemical is baicalin), *Pheretima vulgaris Chen* (hypoxanthine is the major active ingredient), *Stemona tuberosa Lour.* (major compositions are tuber ostemonine, stemonine and croomine), *Aster tataricus L. f.* (major active ingredient is butyl-D-ribuloside), *Ophiopogon japonicus* (L. f.) Ker-Gawl. (major constituents are ophiopogonins), and *Platycodon grandiflorus* (Jacq.) A. DC. (the herb contains platycodins). QQCP possesses prominent effect on relieving cough and reducing sputum [14]. Considering this pharmaceutical properties, we speculated that QQCP might provide an alternative to treat PIC.

To verify the hypothesis, PIC rat model was used to investigate the biological functions of QQCP. Cough frequency and inflammatory cell infiltration of rats were inspected to assess PIC symptoms. Serum and tissue levels of cytokines were tested by ELISA. Histological changes of lung tissue were also examined. In additional, the expressions of substance P (SP), calcitonin gene related peptide (CGRP) and neutral endopeptidase (NEP) in the serum and lung tissue were measured. The findings would provide insights into the clinical use of natural compounds.

## Methods

### Animals

Male Sprague Dawley (SD) rats (200–220 g, 6–8 weeks) were obtained from Heilongjiang Medical University. All animal experiments were carried out in line with National Institutes of Health Guidelines for the Care and Use of Laboratory Animals and approved by the Animal Committee of Heilongjiang Provincial Hospital (KY2017–239). Animals were kept in standard environment (temperature of 25 ± 2 °C, relative humidity of 55–60%,12 h light/dark cycle) and allowed free access to water and food throughout the experiments. PIC rat model was established as previously described [11]. Briefly, rats were exposed to 50 g sawdust and 10 cigarette smokes at 30 min per day for 10 days, followed by instilled intratracheal with lipopolysaccharide (LPS) and capsaicin (10^− 4^ mol/L) aerosols. All animals were randomly grouped into 6 groups (*n* = 10): control, model, low-dose of QQCP (80 mg/kg), medium-dose of QQCP (160 mg/ kg), high-dose of QQCP (320 mg/ kg) and Asmeton group (160 mg/kg) as positive control. Control and model groups were administered with saline for 10 days, other groups were given drugs by gavage for 10 days. Cough frequency of rats was observed continuously twice a day for 10 days after drug administration. At the predetermined times, animals were euthanized with an intraperitoneal injection of sodium pentobarbital at dose of 100 mg/kg to loss of consciousness and control of pain.

### Cell counts in BALF

The right lungs were ligated and the left lungs were lavaged with PBS, then BALF (broncho - alveolar lavage fluid) was immediately centrifuged at 4 °C, 300 g for 10 min. Cell counts were recorded for cell pellets of BALF using blood counting instrument (Invitrogen, US) [15]. A total of 200 cells per slide was counted to forecast the differential cell counts (magnification, 40×). Each cell number was calculated by total number of cells in BALF and the ratio of cell type.

### ELISA assay

The serum and BALF samples were collected to analyze the release of cytokines. Serum and BALF were centrifuged at 4 °C, 3000 g for 15 min and 4 °C, 300 g for 10 min respectively. The levels of TNF-α, IL-1β, IL-6, IL-8, IL-10, IL-13, SP, CGRP and NEP were measured by ELISA kits (CUSABIO, Wuhan, China) according to the manufacturer’s instruction [13].

### SOD and MDA determination

SOD activity and MDA content were evaluated in the serum and lung homogenates by detection kits (Nanjing Jiancheng Bioengineering Institute, Nanjing, China) according to the manufacturer’s protocols [16].

### H&E staining

In the end, lung and bronchus tissues were fixed in 4% paraformaldehyde, embedded in paraffin and sectioned into 4 μm. Then, the samples were stained with hematoxylin for 10 min and treated with eosin for 5 min at room temperature. Histological images were captured at 200X magnification using an Olympus microscope (Olympus, Janpa).

### qRT-PCR assay

Total RNA was extracted from lung tissues using TRIzol reagents (Beyotime Biotechnology, Shanghai, China) and reversely transcribed to cDNA with TaqMan one-step reverse transcription (Applied Biosystems, USA). qRT-PCR was carried out on an ABI Prism 7500 (Applied Biosystems, USA) according to the manufactures’ instructions. Relative expression of SP, CGRP and NEP were calculated using 2^−ΔΔ*C*t^ method, β-actin was used as an internal standard. The specific primers were as following (Table 1).

**Table 1:** Primers

	Forward	Reverse
SP	5′-TGGCGGTCTTTTTTCTCGTT-3′	5′-GCATTGCCTCCTTGATTTGG-3′
CGRP	5′-TCCTGGTTGTCAGCATCTTG-3′	5′-CTCAGCCTCCTGTTCCTCCT-3′
NEP	5′-CTCCCTTCCAGAGACTACTATGA-3′	5′-GACTACAGCTGCTCCACTTATC-3′
β-actin	5′-GTGGGCCGCTCTAGGCACCA-3′	5′-CGGTTGGCCTTAGGGTTCAGG-3′

### Immunofluorescence assay

Lung and bronchus tissues were fixed in methanol and washed with PBS. Samples were incubated with anti-Substance P (ab10353), anti-Endopeptidase (ab73409) and anti-CGRP (ab22560) at 4 °C overnight. Next step, these samples were treated with goat anti-rabbit IgG H&L secondary antibody (ab150077). The nuclei were stained with DAPI. Expressions were monitored by laser scanning confocal microscope (Zeiss, Germany) at 200× magnification.

### Statistical analysis

All data was analyzed using GraphPad Prism 5.0 (GraphPad Software Inc., San Diego, CA) and expressed as mean ± SD. One-way analysis of variance (ANOVA) followed Tukey’s post-hoctest was used to compare differences among multiple groups. *P* < 0.05 was considered as statistically significant.

## Results

### Cough frequency

**Fig. 1:**
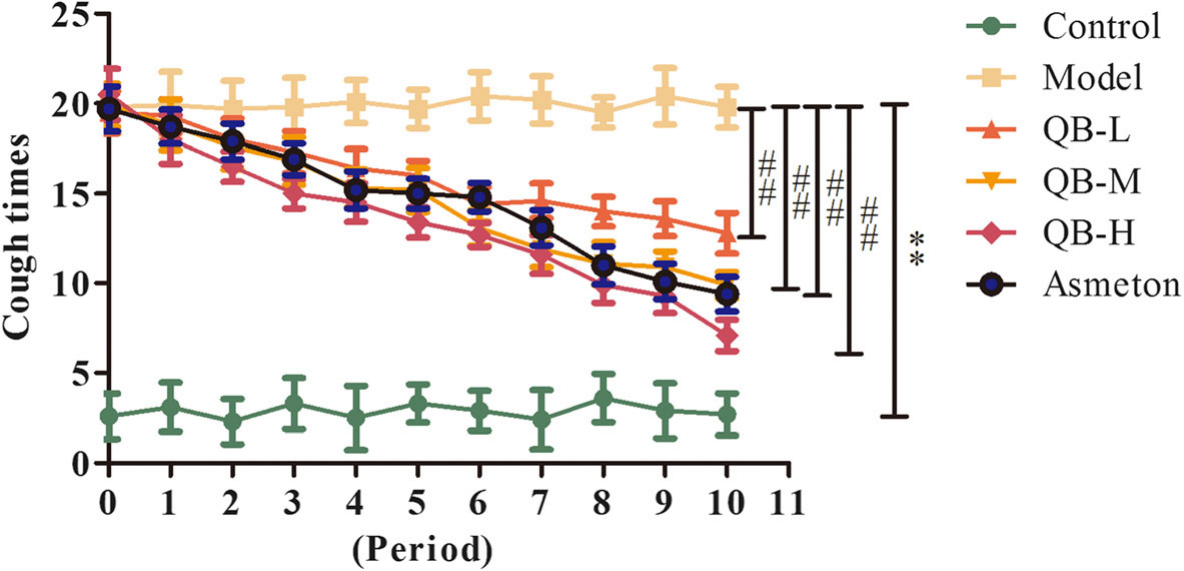
Cough frequency was monitored twice a day for 10 days after drug administration

In order to certify the therapeutic effect of QQCP against PIC, PIC rat models were prepared. Initially, we examined the effect of QQCP on cough frequency of PIC rats. As shown in Fig. 1, during the whole experimental period, CS exposure and LPS administration rats developed durable cough compared with normal rats, unveiling the successful establishment of PIC model. Furthermore, the cough frequency of PIC rats from P0 to P10 was markedly inhibited by treatment with the positive drug of Asmeton. Strikingly, similar to Asmeton, after treatment with QQCP, the rats experienced a pronounced reduction in cough frequency compared to the model group, and the therapeutic effect was positively associated with QQCP dosage. These results highlighted that QQCP could effectively relieve the symptoms of cough.

### Effects of QQCP on inflammatory cell count in BALF

In order to ascertain the influence of QQCP on inflammatory cell infiltration in BALF, analysis of the total number and types of inflammatory cells were conducted. As depicted in Table 2, we observed a remarkable increase in the total number of leukocytes and the proportion of neutrophils in BALF of PIC rats as compared to those in control group. Furthermore, the percentage of macrophages was decreased. QQCP treatment caused a pronounced reduction in the total number of leukocytes in BALF as well as the proportion of neutrophils, but elevation in the proportion of macrophages. Notably, the alterations were most obvious in the rats receiving high-dose QQCP. Nevertheless, the infiltration of lymphocytes cell was negligible. In addition, compared with the positive group, the total number of leukocytes and percentage of neutrophils in QQCP high-dose group were declined, however there was no statistically significant difference between QQCP low/medium-dose group and Asmeton group. The available evidence manifested that QQCP had an inhibitory role on the inflammatory cell infiltration into the lung induced by PIC.

**Table 2:** Effect of QQCP on inflammatory cell count in BALF. Total number of leukocytes and the percentage of neutrophils, macrophages and lymphocytes in BALF were measured

BALF cell count				
	Total number of Leukocytes(× 10^6^)	Neutrophils(%)	Macrophages(%)	Lymphocytes(%)
Control	4.18 ± 0.47	10.28 ± 0.69	83.69 ± 0.47	6.02 ± 0.21
Model	13.5 ± 1.12^**^	22.05 ± 1.65^**^	64.44 ± 2.79^**^	13.51 ± 1.39^**^
QB-L	10.46 ± 0.79^##^	20.8 ± 0.63^##^	67.56 ± 1.03^##^	11.64 ± 0.40^##^
QB-M	8.36 ± 0.74^##^	18.27 ± 0.31^##^	72.56 ± 0.3^##^	9.17 ± 0.18^##^
QB-H	5.92 ± 0.25^##^	15.11 ± 0.27^##^	77.88 ± 0.16^##^	7.02 ± 0.11^##^
Asmton	6.3 ± 0.80^##^	16.93 ± 0.18^##^	72.72 ± 0.83^##^	10.35 ± 0.65^##^

### Effects of QQCP on serum levels of inflammatory cytokines

**Fig. 2:**
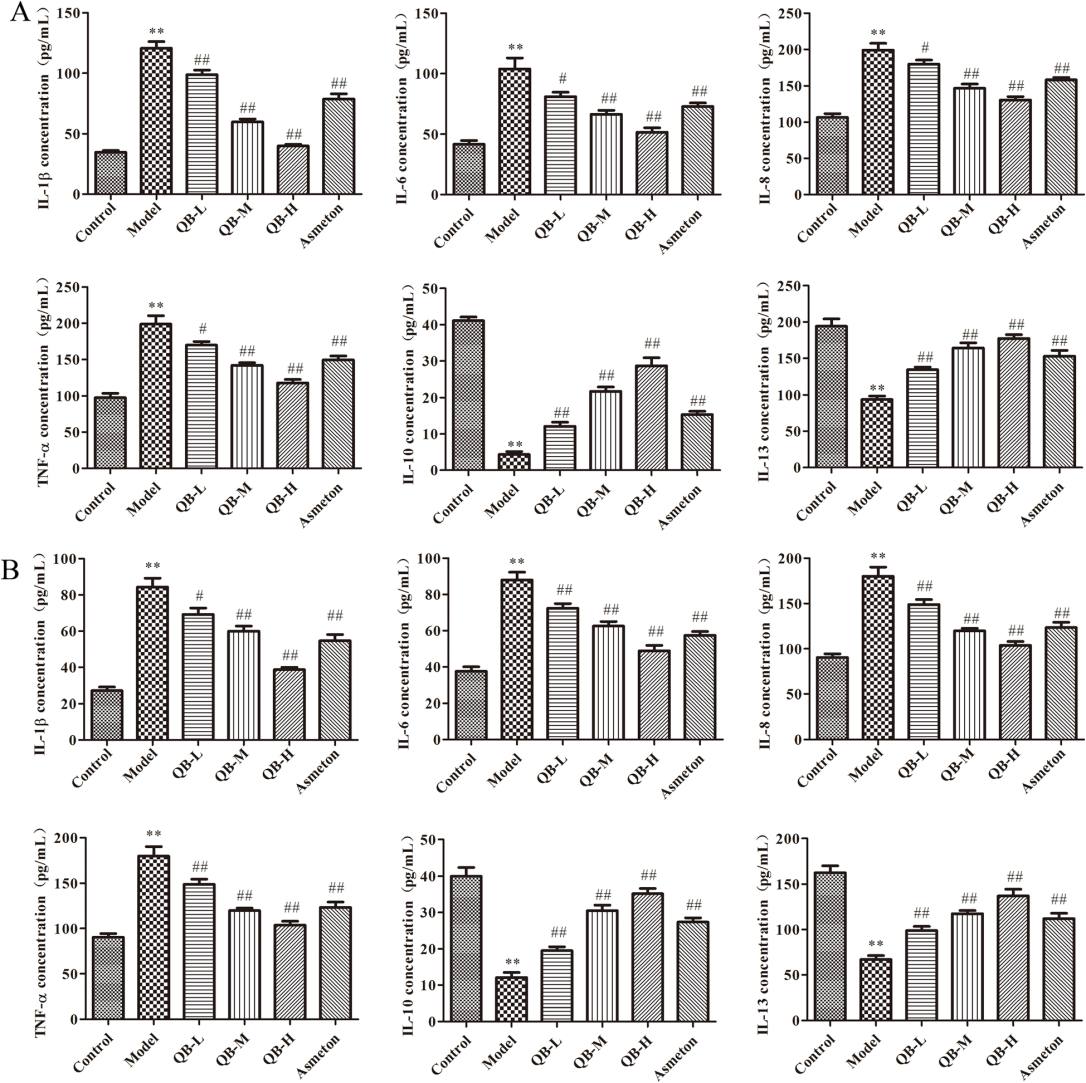
Effect of QQCP on inflammatory cytokines. **a** The levels of TNF-α, IL-1β, IL-6, IL-8, IL-10 and IL-13 in the serum was determined by ELISA. **b** The levels of TNF-α, IL-1β, IL-6, IL-8, IL-10 and IL-13 in BALF was determined by ELISA

To investigate the anti-inflammatory effect of QQCP on PIC, the expressions of cytokines mainly TNF-α, IL-1β, IL-6, IL-8, IL-10 and IL-13 in the serum and BALF were measured. As shown in Fig. 2a and b, the release of pro-inflammatory cytokines (TNF-α, IL-1β, IL-6 and IL-8) were sharply increased in the serum and BALF of PIC rats, while the secretion of anti-inflammatory cytokines (IL-10 and IL-13) were obviously decreased. On the contrary, QQCP and Asmeton dramatically blocked the inflammatory response, leading a decrease in the expression of pro-inflammatory molecules TNF-α, IL-1β, IL-6, IL-8 and an elevation in anti-inflammatory molecules IL-10 and IL-13, while QQCP at high concentration was more effective in preventing the inflammation. These outcomes suggested that QQCP could attenuated PIC-induced inflammatory response in a dose-dependent manner.

### Effects of QQCP on SOD and MDA in serum and lung tissue

**Fig. 3:**
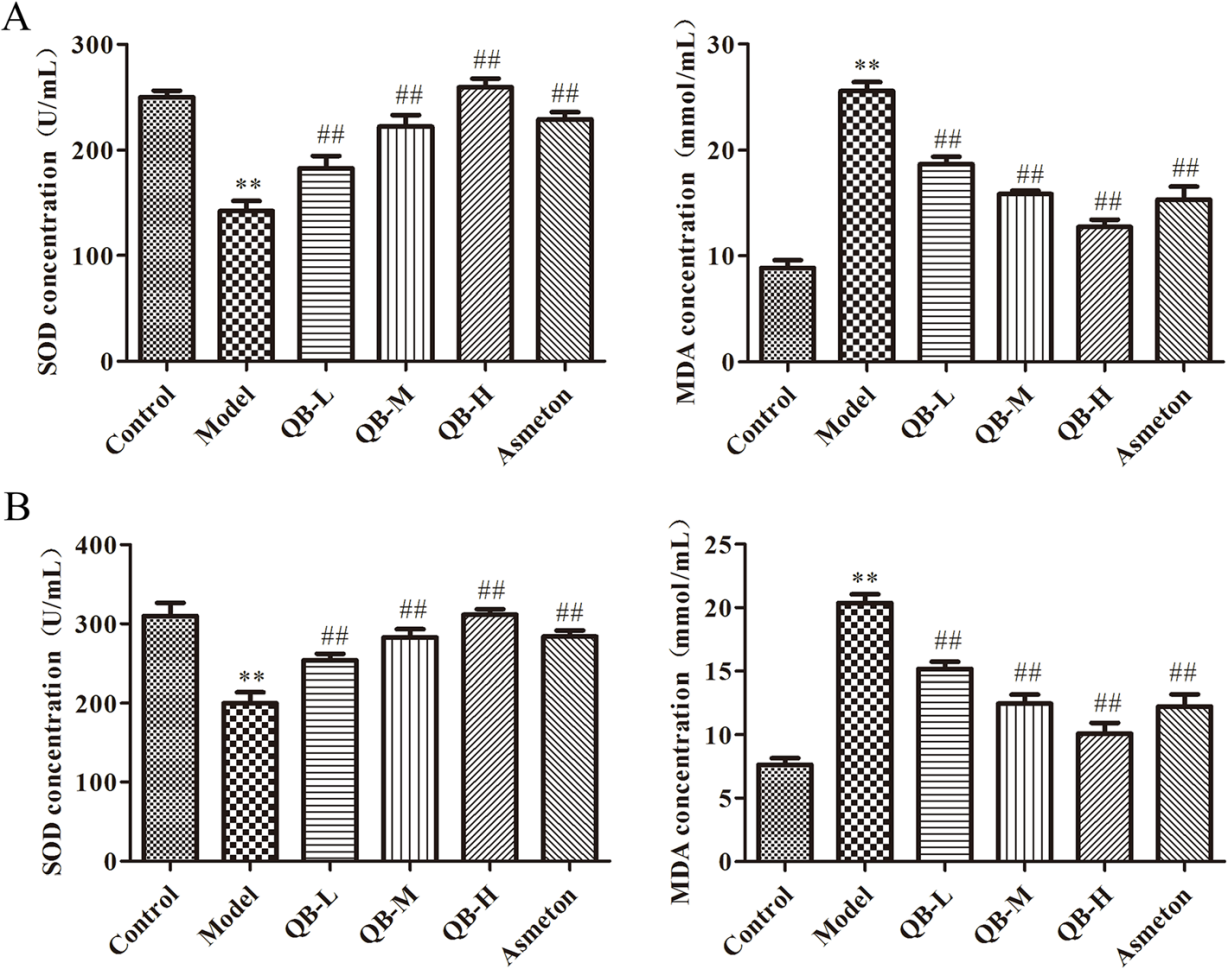
Effect of QQCP on oxidative stress in the serum and lung tissue. **a** SOD and MDA activities in the serum were detected by ELISA. **b** SOD and MDA activities in the lung tissue were detected by ELISA

To explore whether QQCP played impacts on oxidative stress, the activity of oxidative stress indicator MDA and antioxidant enzyme SOD in the serum and lung tissue were examined. As presented in Fig. 3a and b, CS exposure and LPS treatment resulted in prominent decrease of SOD activity in the serum and lung tissue when compared with the control group, indicating that oxidative stress was prompted in the development of PIC. Encouragingly, QQCP treatment dose-dependently improved SOD activity in the serum and lung tissue. Meanwhile, MDA content in the serum and lung homogenate was higher in PIC model group than control group, while QQCP and Asmeton decreased the content of MDA observably. However, the differences in SOD activity and MDA content between QQCP low/medium and Asmeton groups were not statistically significant. These data demonstrated that QQCP could efficiently counteract the oxidative damage during the progress of PIC.

### Lung histopathology analysis

**Fig. 4:**
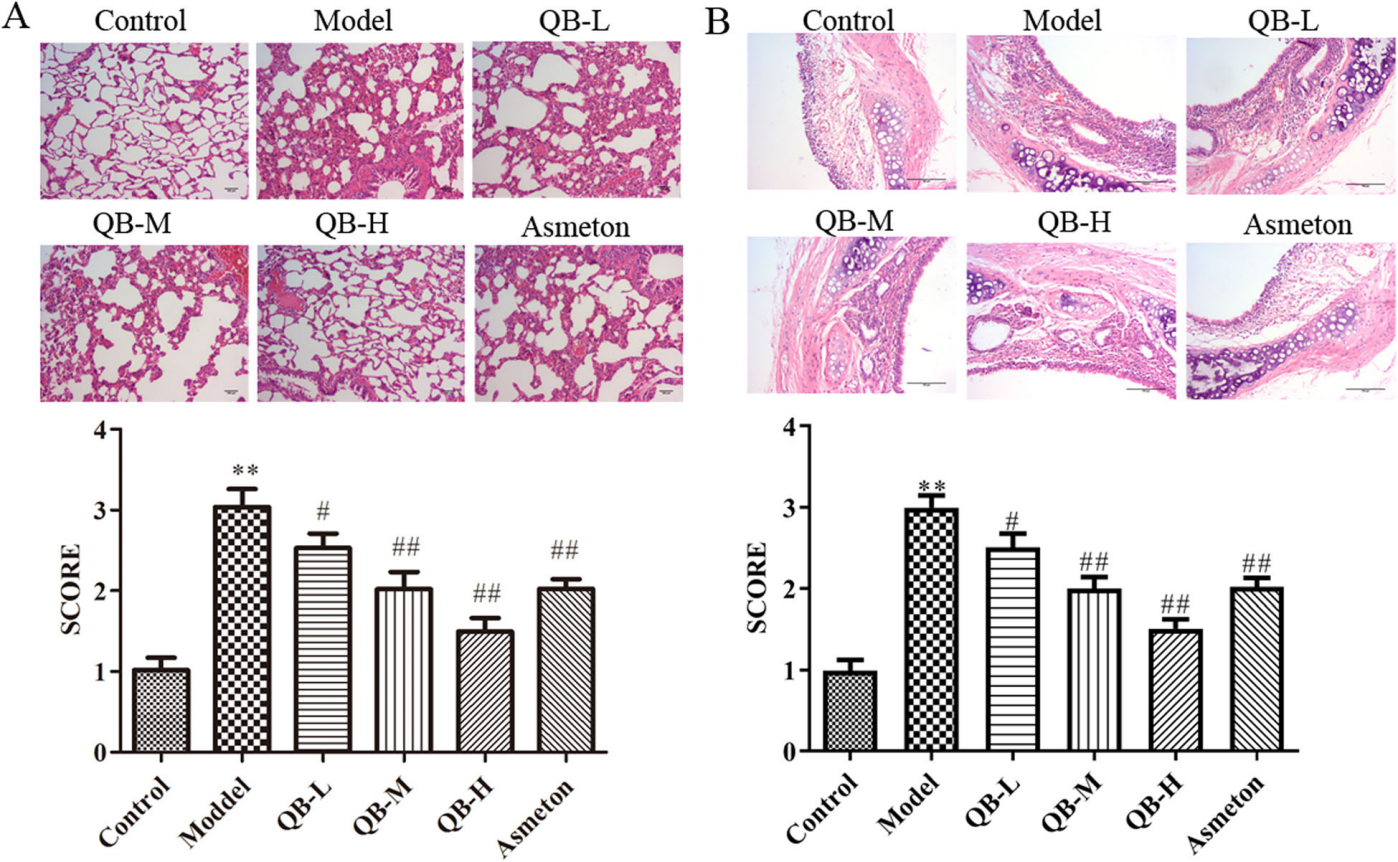
Effect of QQCP on histological changes of tissues. **a** The histopathology of lung tissue in rats was evaluated by H&E staining. **b** The histopathology of bronchus tissue in rats was evaluated by H&E staining

To observe the histopathological changes in the lung and bronchus, H&E staining was performed. As displayed in Fig. 4a, the lung tissue of healthy rat was characterized by a thin alveolar wall with scarce inflammatory cells. However, histopathological changes in the lung of PIC rat comprised marked inflammatory responses such as thickened alveolar walls, inflammatory cell infiltration and a formatted hyaline. The similar outcomes of histological changes in bronchus tissue were shown in Fig. 4b. Compared with PIC model group, these histological damages were significantly ameliorated by either QQCP or Asmeton, which could further support the results of cell count in BALF. More specially, the histological lesion decreased as QQCP concentration increased, but no significant difference in histological changes was observed in QQCP low/medium groups and Asmeton group. These data clearly indicated the importance of QQCP in regulating the inflammatory aspect of this model.

### Effects of QQCP on SP, CGRP and NEP in BALF, serum and lung tissue

**Fig. 5:**
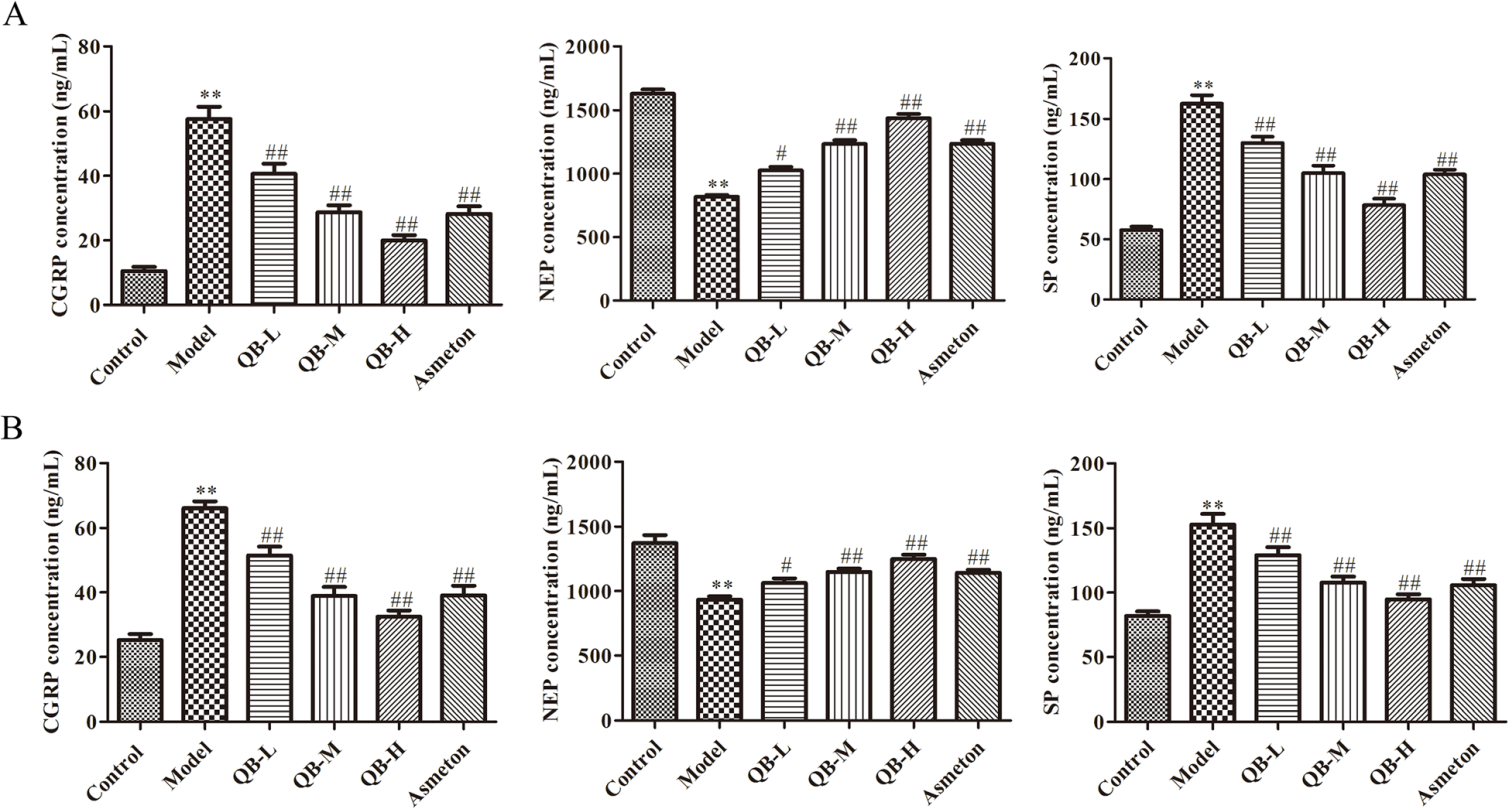
Effect of QQCP on neurogenetic inflammation in BALF and serum. **a** SP, CGRP and NEP levels in BALF were measured by ELISA. **b** SP, CGRP and NEP levels in the serum were measured by ELISA

**Fig. 6:**
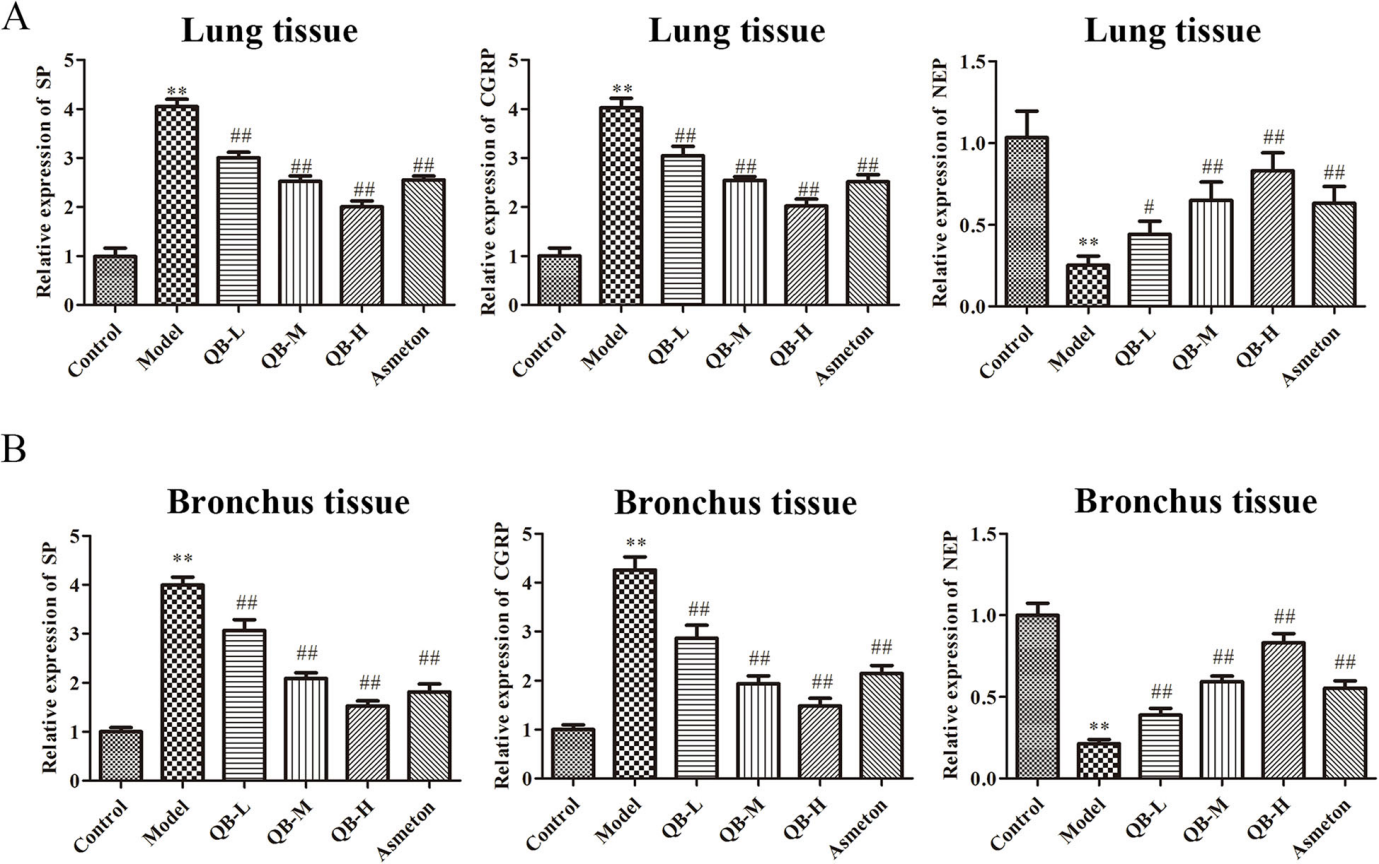
Effect of QQCP on neurogenetic inflammation in the lung tissues. **a** SP, CGRP and NEP levels in the lung tissue were measured by qRT-PCR. **b** SP, CGRP and NEP levels in the lung tissue were assessed by immunofluorescence

**Fig. 7:**
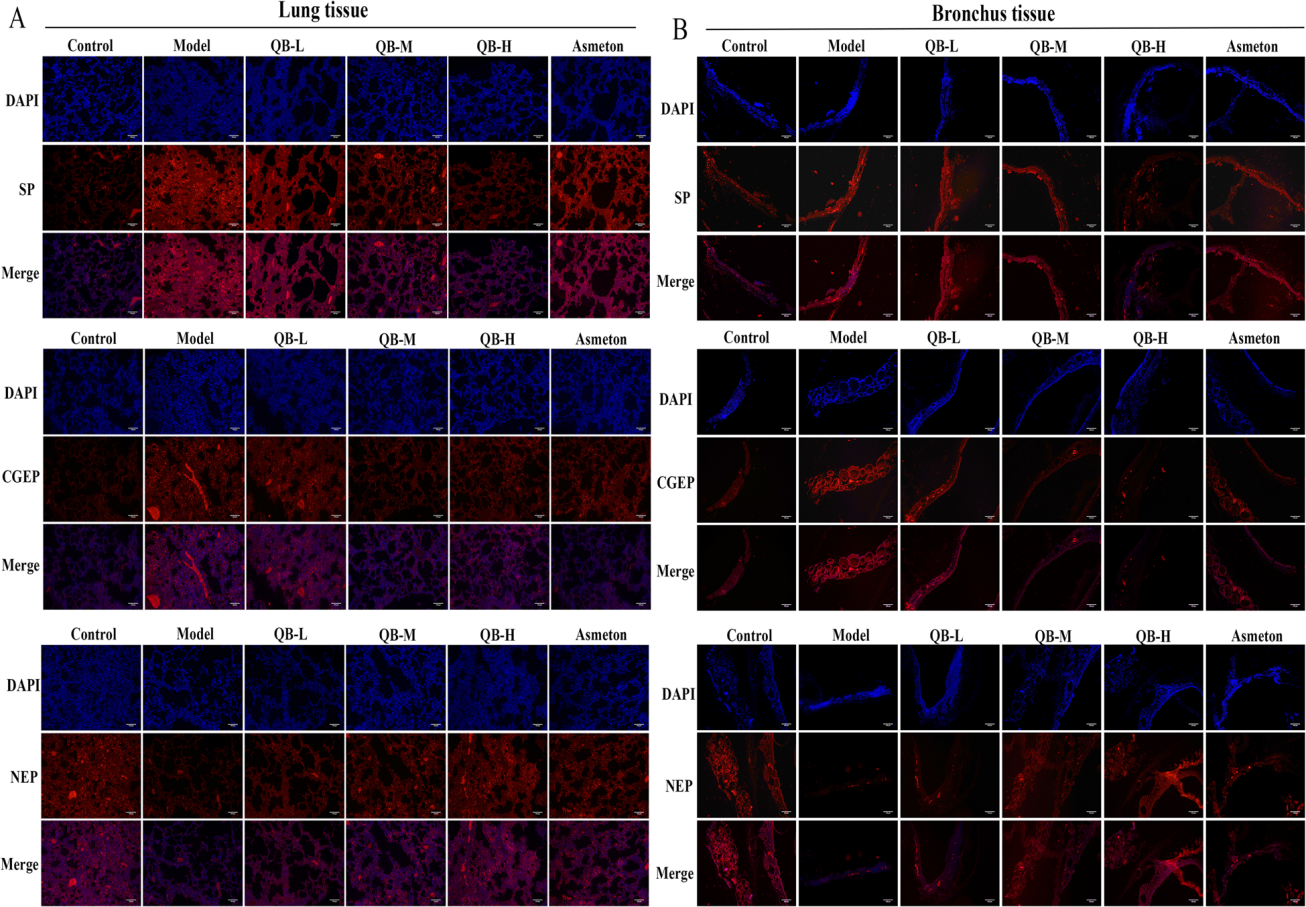
Effect of QQCP on neurogenetic inflammation in the bronchus tissues. **a** SP, CGRP and NEP levels in bronchus tissue were measured by qRT-PCR. **b** SP, CGRP and NEP levels in bronchus tissue were assessed by immunofluorescence

It has been proposed that expression of neuropeptides is altered when challenged with inflammation or injury [17]. Thus, levels of neuropeptides SP, CGRP and NEP in BALF, serum, lung and bronchus tissue were detected at the end of the experiment. In BALF and serum, PIC induced inflammation led to upregulation in the expression of SP and CGRP, and downregulation in NEP level (Fig. 5a and b). However, such changes were partly reversed after QQCP administration at different concentrations. Then, in order to confirm the altered expressions of SP, CGRP and NEP in the lung tissue of rats, we compared the expressions of SP, CGRP and NEP in the lung by qRT-PCR and immunofluorescence assays. As expected, the mRNA expressions of SP and CGRP were sharply raised in the lung, and NEP expression was diminished in PIC rats compared to the control group (Fig. 6a). Following QQCP treatment, the mRNA expressions of SP and CGRP were decreased significantly, whereas the mRNA expression of NEP was enlarged compared with the model group. Parallelly, from the result of immunofluorescence (Fig. 6b), dramatically greater SP and CGRP fluorescence were found in the lung of PIC rats than in that of the normal rats. Notably, the fluorescent signal of SP and CGRP was weakened followed QQCP treatment. In addition, QQCP greatly promoted the expression of NEP as reflected by enlarged NEP fluorescence. In bronchus tissue, within QQCP administration, the neuroinflammation was significantly improved as reflected by increased NEP expression and reduced SP and CGRP levels (Fig. 7a and b). Overall, Our findings all indicated that QQCP had protective effect against the neurogenetic inflammation in the progress of PIC.

## Discussion

Post-infectious cough, defined as sub-acute cough, is commonly associated with upper respiration tract infection. Currently, some complex pathogenesis of cough has been revealed by humans however, there is still no specific drug for PIC. Fortunately, some TCM have been created in improving clinical symptoms of cough with sufficient efficacy [18, 19]. Herein, we clarified that QQCP, a classic prescription of TCM, dose-dependently alleviated PIC by inhibiting cough frequency, inflammatory cell infiltration and oxidative stress responses, neurogenetic inflammation and improving the pathological damages in the lung tissue.

In the current study, to establish PIC model, we treated the rats with CS and LPS for 10 days to mimic gram-negative bacteria exposure. It has been found that LPS, located in the outer membrane of gram-negative bacteria, could provoke powerful immune responses in animals, thereby leading to inflammatory process [20]. We observed that PIC rats developed continuous cough during the entire experimental period. Besides, highly increased levels of leukocytes and neutrophils but a reduction of macrophages in BALF of PIC rats were exhibited, which might enlarge lung lesion via the production of cytokines and chemokines. Consistently, existing research has revealed that CS exposure could affect the immune function of the respiratory system that causes the infiltration of inflammatory cells into the lungs as well as the secretion of inflammatory mediators [21]. To be mentioned, following treatment with QQCP, cough was visibly ameliorated as evidenced by the descend in the number of total cells and the percentage of neutrophils in BALF. There has been previous study regarding the therapeutic effect that QQCP has significant activity in preventing and treating *M.pneumoniae* infection [22].

It is well accepted that dysregulated inflammation is a hallmark of lung diseases [23]. Previous research has suggested that both anti- and pro-inflammatory mediators (eg. TNF-α, IL-6, IL-8 and IL-10) exert an important role in initiating and maintaining the inflammatory process in lung injury [24]. Inflammatory cytokines can alter the permeability of alveolar epithelial cells and cause activated inflammatory cells infiltration, which ultimately result in tissue lesions [25]. In our investigation, we discovered that QQCP exhibited significant suppression of pro-inflammatory makers and promotion of anti-inflammatory markers during the process of PIC. The results were in line with inflammatory cell infiltration in BALF since inflammatory cells especial neutrophils could secret inflammatory factors such as TNF-α, IL-1β and IL-6 [26]. Given that the production of inflammatory molecules like TNF-α and IL-6 are associated with the severity of histological lesion in the lung [27, 28]. We further conducted the histological analysis and found that QQCP attenuated the lung and bronchus damage caused by PIC as reflected by declined inflammatory cells recruitment. These results implied that QQCP attenuated PIC towards a less inflammatory state and simultaneously enhanced the bioregeneration of damaged tissues.

In addition to inflammatory molecules, mounting evidence suggest that oxidative stress greatly influence the degree of lung injury [16]. Oxidative stress, induced by an imbalance between the oxidative and antioxidative stress response, is mainly triggered by deletion of SOD and excessive MDA accumulation [29]. As one of the most important antioxidant enzymes, SOD can catalyze the dismutation of superoxide anions to form hydrogen peroxide. MDA, one of the lipid peroxidation products, represents the extent of lipid peroxidation. In our investigation, SOD and MDA levels were measured in the serum and lung tissue to assess the impact of QQCP on oxidative stress. The findings indicated that PIC led to declined SOD activity and elevated MDA level, which could be offset by QQCP. Hence, we could infer that the protective function of QQCP against PIC was partially attributed to the maintenance of balance between oxidative and antioxidative responses.

More importantly, it is commonly believed that neurogenic inflammation has been involved in the pathogenesis of PIC [11]. NEP, also known as CD10, which is the primary degrading enzyme of neuropeptides in airway that hydrolyzes neuropeptides such as SP and CGRP [30]. Excessive neuropeptides expression and impaired NEP activity in the lung have been testified in respiratory viral infection and pulmonary diseases [31]. In our study, the presence of decreased NEP activity accompanied by elevated SP and CGRP expression in BALF, serum, lung and bronchus of PIC rats might have resulted in neurogenic inflammation, which could explain the associated symptoms. Notably, the inflammation was dramatically abolished as confirmed by enhanced NEP activity and reduced SP and CGRP level through QQCP treatment. Based on the above outcomes, the decline in inflammatory cell recruitment, suppression of inflammatory response, oxidative stress and neurogenic inflammation mediated by QQCP, might have potential protective effects against the pathogenesis of PIC.

## Conclusion

In conclusion, we confirmed that QQCP exhibited potential therapeutic properties against PIC by inhibiting inflammatory cytokines, such as TNF-α, IL-1β, IL-6, IL-8.,suppressing oxidative stress and increasing the neuropeptide secretion. The ability of QQCP to protect against PIC might be the consequence of the synergistic anti-inflammatory and antioxidant effects. Thus, our study provides evidence that QQCP may emerge as a therapeutic option for PIC treatment.

## Data Availability

All data generated or analysed during this study are available from the corresponding author on reasonable request.

## Abbreviations

TCM: Traditional Chinese medicine
QQCP: Qinbai Qingfei Concentrated Pellet
SOD: Superoxide dismutase
PIC: post-Infectious cough
MDA: Malondialdehyde
SP: Substance P
CGRP: Calcitonin gene related peptide
NEP: Neutral endopeptidase
BALF: Bronchoalveolar lavage fluid
TNF-α: tumor necrosis factor-α
IL: Interleukin; cigarette smoke (CS)
LPS: lipopolysaccharide
SD: Sprague-Dawley
